# Unveiling the photocatalytic and antimicrobial activities of star–shaped gold nanoparticles under visible spectrum

**DOI:** 10.1038/s41598-024-82332-8

**Published:** 2025-01-07

**Authors:** Ahmed M. El-Khawaga, Amir Elsaidy, Miguel A. Correa-Duarte, Sherif Elbasuney

**Affiliations:** 1 Department of Basic Medical Sciences, Faculty of Medicine, Galala University, New Galala City, Suez Egypt; 2 School of Chemical engineering, Military Technical College, Cairo, Egypt; 3 Department of Physical Chemistry, Biomedical Research Center (CINBIO), Institute of Biomedical Research of Ourense-Pontevedra-Vigo (IBI), Universidad de Vigo, 36310, Vigo, Spain; 4 Head of Nanotechnology Research Center, Military Technical College, Egyptian Armed Forces, Cairo, Egypt

**Keywords:** Gold nanostars, Water treatment, Photocatalysis, Antimicrobial activity, Environmental chemistry, Nanobiotechnology

## Abstract

This study reports on the facile development of star-shaped gold nanoparticles via seed-mediated growth protocol. Gold nanostars (AuNSTs) demonstrated average particle size of 48 nm using transmission electron microscopy (TEM). Chemical composition of AuNSTs was verifired using energy dispersive X-ray spectroscopy (EDX) mapping. AuNSTs demonstrated high optical response under visible spectrum, with maximum absorption at 685 nm, using UV-Vis spectroscopy. Therefore AuNSTs could be involoved into photocatalytic reaction under visible spectrum. AuNSTs demonstrated superior performance in degradation of rhodamine B dye (RB), and disinfection of some pathogenic bacteria. AuNSTs offered enhanced removal efficiency against rhodamine B dye (82.0 ± 0.35% in 135 min) under visible irradiation. Remarkably, under proper conditions of pH = 9, approximately 94 ± 0.55% of a 10 ppm RB solution was effectively photodegraded after 135 min; this could be ascribed to the strong electrostatic attraction between negatively charged AuNSTs surface and positive RB contaminant. This superior photocatalytic activity of AuNSTs could be correlated to high interfacial charge transfer efficiency for Au, and enhanced charge pair separation under visible spectrum. Additionally, AuNSTs exhibited potential antibacterial activity against *Escherichia coli* (*E. coli*) and *Staphylococcus aureus* (*S. aureus*). AuNSTs demonstrated substantial antibacterial activity via disk diffusion and microbroth dilution tests with zones of inhibition and minimum inhibitory concentrations (MIC) for *E. coli* (20.0 ± 0.54 mm, 1.25 µg/ml) and *S. aureus* (23.0 ± 0.35 mm, 0.625 µg/ml), respectively. In conclusion, AuNSTs demonstrated efficient dye removal capabilities along with significant antimicrobial activity against gram-positive and gram-negative bacterial strains.

## Introduction

The hierarchical structure of marine starfishes and sea urchins has been the basis or core of new nanosized materials. Such nanostructures can offer multiple rays of rams extended through a core body, which enlarge the surface to volume ratio, active surfaces, and adsorptive surfaces^1,2^. One of the outstanding members of these novel nanostructures is star-shaped nanoparticles (NPs) known as nanostars. Gold nanostar (AuNST) is the most extensively investigated nanomaterial in the field of nano biotechnology^3^. Star-shaped colloids offer extraordinarily high electromagnetic fields at their tips; they can be used as optical enhancers. Enhanced colloidal stability could withstand significant catalytic activity. AuNSTs have attracted wide attention due to their potential applications in catalysis^4^, antimicrobial^5^, anticancer^6^, drug delivery^7^, and agriculture^8^. In this regard, the optical properties, for instance, localized surface plasmonic resonnance (LSPR), surface-enhancement Raman Scattering (SERS) and catalytic properties, are the main outcomes and features that gold nanostars have grabbed considerate attentions particularly in water treatment applications. For example, they can play an essential role as photosensitizers, since able to enhance the photocatalytic activities of large bandgap semiconductors in a broader electromagnetic spectrum^9^.

In this context, water is the essential sources for life. However, different type of dyes like methylene blue, Phenolic Azo Dyes, Alizarin S, Crocein orang, methyl red, and other organic pollutants elute from industries in the form of waste matter^10,11^. These contaminants expose severe threat to all kind of living organisms^12,13^. In the textile industry, Rhodamine B (RB) is a highly water-soluble xanthene organic dye, which is widely used in the plastics, textile industries, ceramics sector, paper industry and analytical chemistry^14^. Nonetheless, due to industrialisation and unlawful discharge of the RB; it poses health risks to humans and wildlife that enters the food chain^15^. Therefore, food for human consumption should be routinely monitored and strictly regulated for the risks of RB contamination. European Food Safety Authority (EFSA) declared RB to be potentially genotoxic and carcinogenic^16^. Consequently, the use of RB in food stuffs is forbidden in all EU and many other countries^17^. Interestingly, biosynthesised metal-based nanoparticles such as silver (Ag) and gold nanoparticles (AuNPs) experienced wide biomedical applications^18,19^. Recently, gold NPs have been used as antibacterial agents against broad range of micro-organisms. Their great impact, bio-compatibility, low cost, and facile production are some advantages of gold nano structures^20,21^. Numerous reports have described the production approaches of star-shaped gold nanostructure^22–24^. Synthetic protocols are classified into two main groups: seedless and seed-mediated growth methods. In this consideration, seed-mediated method, recogenized as two stage strategy, can offer better control over particle size and shape^25^. This study reports on facile synthesis of gold nanostars (AuNSTs) and the evaluation of their antimicrobial activity and photocatalytic in the degradation of rhodamine B (RB) dye. The findings reveal that the synthesized AuNSTs is a promising photocatalyst since it offered enhanced degradation efficiency of RhB by approximately (94 ± 0.55%) after 135 min, and exhibited significant antimicrobial activity against *E. coli* (ZOI: 20.0 ± 0.54 mm) and *S. aureus* (ZOI: 23.0 ± 0.35 mm).

## Experimental section

### Chemicals

Tetrachloroauricacid (99.9%, HAuCl_4_.3H_2_O), trisodiumcitrate (98%, Na_3_C_6_H_5_O_7_), (polyvinylpyrrolidone) (PVP, Mwt 10k), N,N dimethylformamide DMF (99%), ethanol (99.5%) and rhodamine B (RB) (> 99%) were purchased from Sigma-Aldrich. Milli-Q grade water were used in all preparation. All chemicals were used without further purification.

### Synthesis of PVP-coated gold seeds

**Fig. 1:**
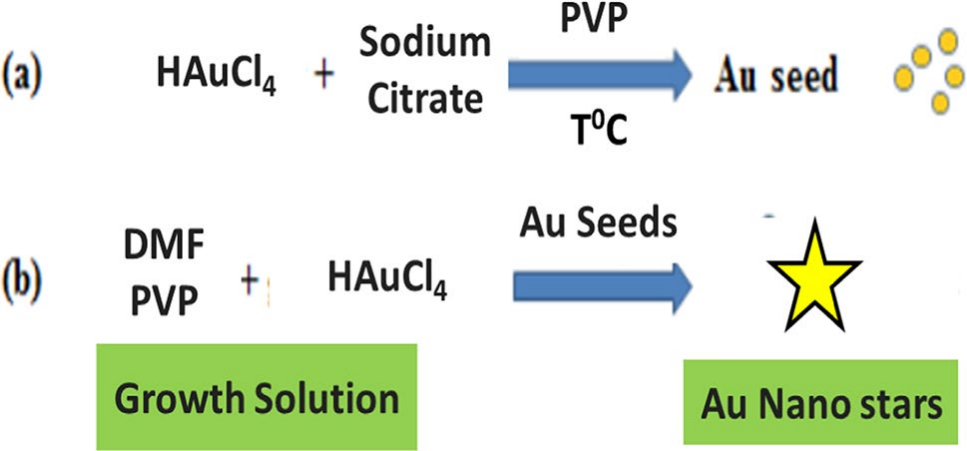
A schematic diagram of synthesis protocol of AuNSTs.

Gold seeds were synthesized via established reported seed-mediated method with modifications^26,27^ (Fig. 1). Brifely, 2.5 mL of a 0.034 M sodium citrate aqueous solution was introduced to 47.5 mL of a boiling 0.5 mM HAuCl_4_ aqueous solution while maintaining vigorous stirring. The boiling mixture was allowed to react for one hour under stirring. During this time, the color of the solution gradually changed from colorless to red. Subsequently, 5 mL of an aqueous polyvinylpyrrolidone (PVP, 10k) solution (0.03 g/mL) was added dropwise to the aqueous gold seed solution under stirring. The mixture was left under stirring for a day at room temperature to maximize PVP adsorption.

The PVP-coated gold seeds were subjected to centrifugation five times at (4500 rpm, 45 min). In each washing step, the collected nanoparticles were redispersed in ethanol, and the supernatant was retained for centrifugation until it became colorless. Ultimately, the seeds were redispersed in ethanol with final concentration of 3.42 mM.

### Synthesis of PVP-coated gold nanostars

AuNSTs were developed through a previously reported modified synthetic procedures^27,28^ (Fig. 1) .In particular, 1.5 g of PVP (10 K) were dissolved into 15 mL of DMF. After its complete dissolution, the mixture was further sonicated for 30 min. Then, 33.86 µL of a HAuCl_4_ aqueous solution (0.1196 M) were added to the solution, followed by the injection of 105.26 µL of PVP-coated gold seeds in EtOH (0.0034 M) under stirring.The solution was let for two hours in an ice bath under stirring. Within 20 min the color of the solution was changed to blue, indicating the formation of Au nano stars. The acquired colloids were submitted to 3 times centrifugation steps at (4500 rpm for 45 min) to remove DMF and excess of PVP. In all the steps the particles were resuspended in ETOH (10 ml). At the end, the harvested nanomaterials redispersed in EtOH for further characterizations.

### Characterization of gold nanostars

Transmission electron microscopy (TEM) measurements were performed on a JEOL JEM 1010 instrument operating at an acceleration voltage of 100 kV, with a CCD camera. Samples for the TEM analysis were prepared by dropping a diluted suspension of AuNSTs onto an ultra-thin carbon-coated copper grids. The optical response of developed gold nanostars was recorded on a Cary 8454 UV-visible-NIR spectrophotometer. Elemental mapping by EDS analysis were carried out with HRTEM FEG JEOL 2010 F operating at an acceleration voltage of 200 kV.

### Photocatalytic activity of gold nanostars

**Fig. 2:**
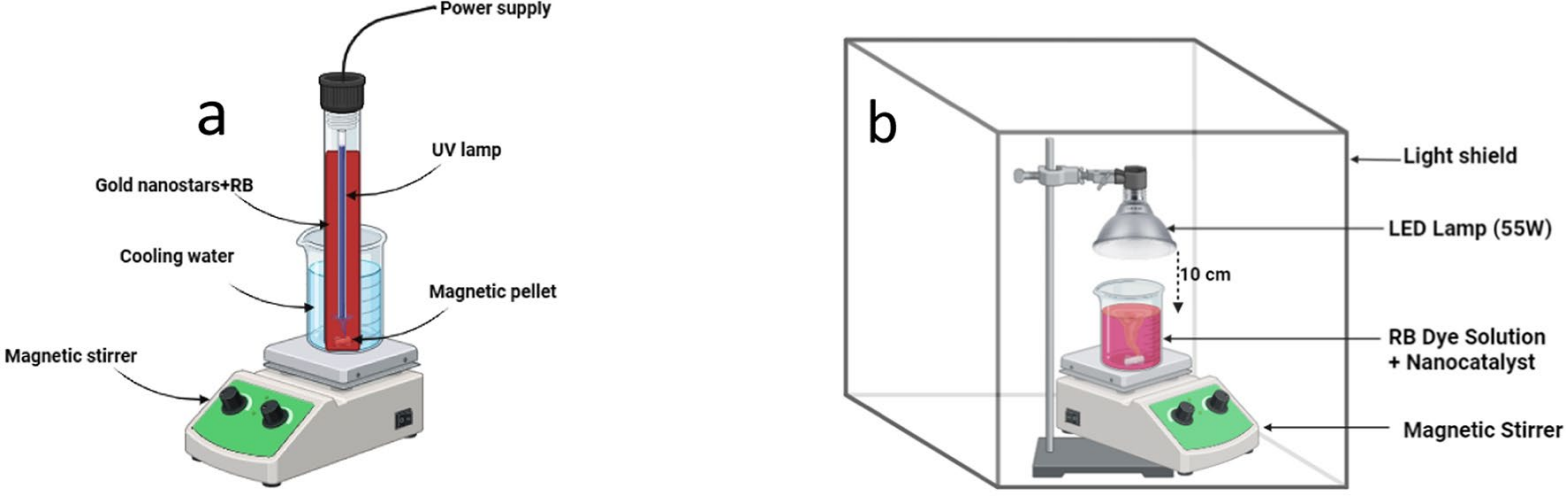
The photocatalytic setups for UV irradiation (**a**), Visible light irradiation(**b**).

Gold nanostar sample of 10 mg was introduced into 50 ml aqueous solution containing RB dye with an initial concentration (C_0_) of 10 mg/L. The mixture was stirred continuously at room temperature (25 °C) for 30 min in the absence of light to set equilibrium between adsorption and desorption processes. Subsequently, a simulated UV light source in the form of a UV lamp was employed to illuminate the solution containing the photocatalyst and RB. The position of employed UV lamp was setted axially within aquartz immersion tube. At fixed time intervals over irradiation, a syringe equipped with a filter (pore size of 2.5 μm) was employed to extract a 1 ml sample of the RB suspension. The source of ultraviolet (UV) light irradiation for this experiment was a commercially available Philips TUV 11WG11 T5 UV-C lamp, which is a high-pressure mercury vapor lamp emitting radiation at a mean wavelength of 254 nanometers. This lamp was submerged within the contaminated solution, while the photoreactor was maintained at a temperature of approximately 15 ^o^C through the use of a cold-water bath. In contrast, the visible light employed for irradiation was generated by a custom-built apparatus consisting of 52 white LEDs with a nominal power output of 55 watts and an emission spectrum spanning the range of 400–800 nanometers. For minimizing losses due to irradiation, the LEDs were surrounded by aluminum reflectors. The irradiation was applied from above, with a fixed distance of 10 centimeters separating the light source from the photoreactor as represented in Fig. 2 (a and b). In this context, the removal rate of RB was determined by assessing the variation in dye concentration over the course of irradiation time using a UV-visible spectrophotometer (Agilent Technologies Cary 60 UV-visible) at a wavelength (λmax) of 546 nm. Deionized water served as the reference medium^29^.

Firstly, under continuous stirring the RB dye and gold nanostars were placed into the glass cylindrical reactor under UV irradiation, then, Aliquots of 1 mL are taken within 30 min intervals during the experiments in order to measure the variation in the absorbance of the dye that was conducted via aspectrophotometer at specific wavelength of 546 nm^30^. The photodecomposition efficiency (Removal %) was estimated using the following formula^31^:

1 $${\text{Removal }}\% =1 - \left( {\frac{{Ct}}{{C0}}} \right)*100$$

Where C_o_ is the contaminant’s initial concentration (in mg/L), and C_t_ represents the concentration at a given time (t).

The operational parameters of photocatalytic degradation, including initial pollutant concentration and pH, were addresed.

### Antimicrobial activity of AuNSts

The antimicrobial effectiveness of the AuNSts was evaluated through agar-disc diffusion technique^32^, which involves testing ability of AuNSTs to inhibit the growth of various microorganisms^33^. The size of inhibition zone is usually related to the level of antimicrobial activity present in the sample; larger inhibition zone means more potent antimicrobial agent. The measurement of inhibition zone was conducted by using a physical ruler like a meter scale. The scale was placed above the petri-dish and the diameter was recorded^34^.

This study assessed the inhibitory effects of AuNSts on both Gram negative *E. coli* (ATCC 25922) and Gram positive *S .aureus* (ATCC 25923). Control samples consisting of conventional antibiotic discs impregnated with gentamicin (CN, at a concentration of 10 µg per disc) and measuring 6.0 millimeters in diameter were also included for evaluation purposes. In this regard, the minimum inhibitory concentrations (MIC) of the most effective antimicrobial specimens were quantified through the application of the serial dilution method within a Luria-Bertani (LB) agar-based growth medium^32^. Both positive and negative controls were employed in this approach; the positive control contained both the growth medium and the pathogenic bacteria. Furthermore, the generated nanoparticles were incorporated into the evaluation procedure, initially at a concentration of 20.0 µg/mL. After incubation at a controlled temperature of 36.0 ± 1.0 °C for1 day, the MIC values were determined^35^.

## Results and discussions

### Characterization of gold nanostars

**Fig. 3:**
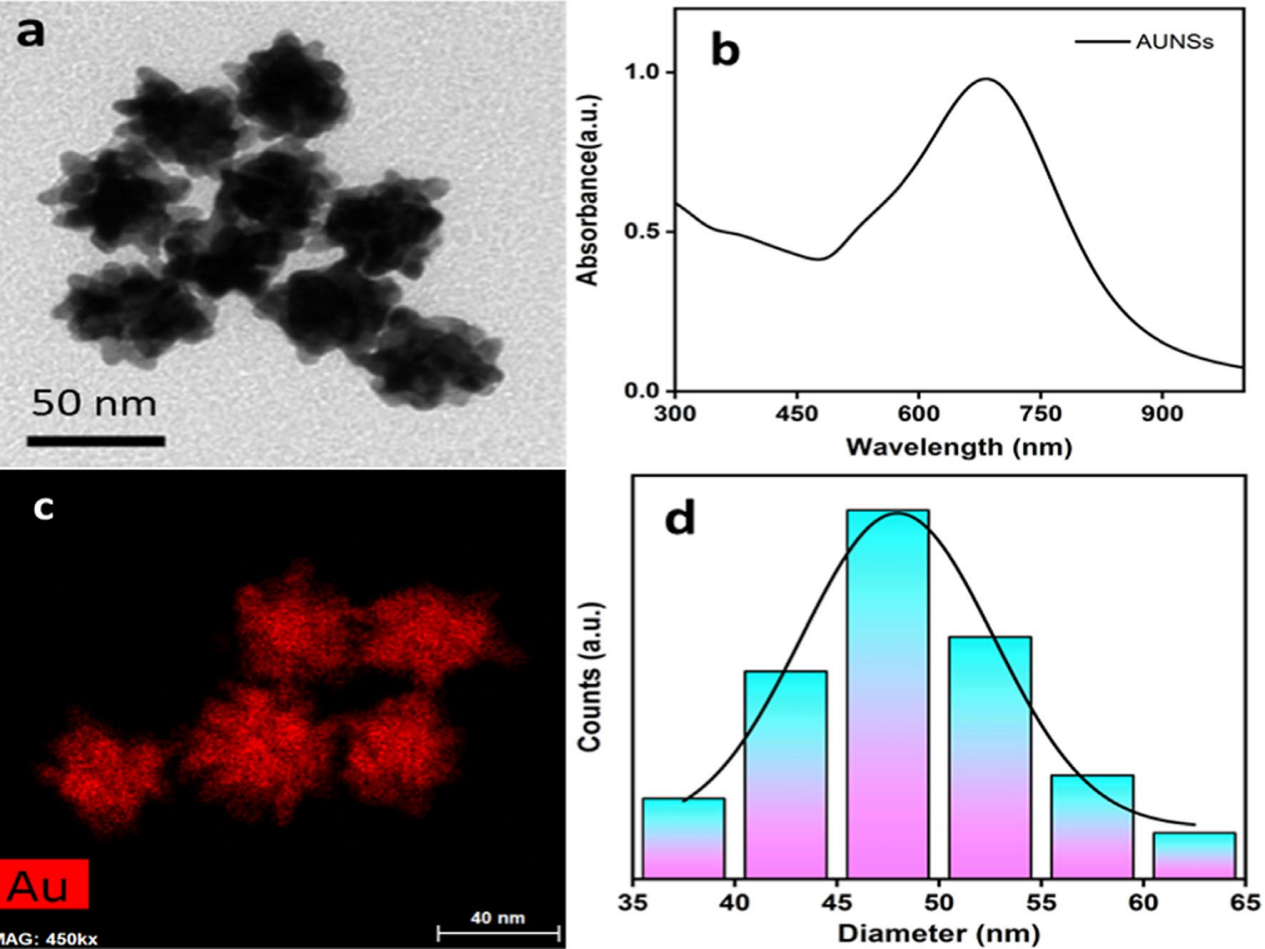
TEM micrograph of AuNSTs (**a**), UV-vis absorption spectrum of AuNSTs with a major plasmonic resonance centered at 685 nm (**b**), Energy dispersive X-ray (EDX) analysis of AuNSTs (**c**) and particle size distribution analysis of the synthesized AuNSTs (**d**).

Gold nano stars (AuNSTs) with average diameter 48 ± 9 nm were synthesized via controlled seed- mediated growth strategy. Such strategy can provide a temporal separation between seeds and nucleation, since seed nanoparticles are synthesized independently and then introduced into a growth solution. As aconsequence, better control over shape and size can be accomplished^36^. The morphological analysis of the obtaind nano stars has been carried out by transmission electron microscopy (TEM) (Fig. 3a). The optical response shows the two characteristic bands that match the core (shoulder at 550 nm) and outer spikes (broad band centered at 685 nm) for the prepared nanostructures (Fig. 3b).Gold nanostars typically consist of a solid plasmonic core, like a sphere, which supports protuberant plasmonic tips. The core provides electrons to the tips, and strengthen the electric field as it grows^37^. Regarding the importance of the tips (outer spikes), the main plasmonic features of these nano structures arise from the tips that concentrate electromagnetic fields in small regions (hot spots), while offering great efficiency in optical sensing, environmental research, biology, and medicine. Energy dispersive X-ray spectroscopy (EDX) mapping was carried out for assessing the composition of the acquired nano stars Fig. (3c). Particle size distribution analysis has been conduted (Gaussian fit) for the obtained gold nano stars with average diameter of 48 ± 9 nm from tip to tip length (Fig. 3-d).

### Removal performance of AuNSTs against RB dye

**Fig. 4:**
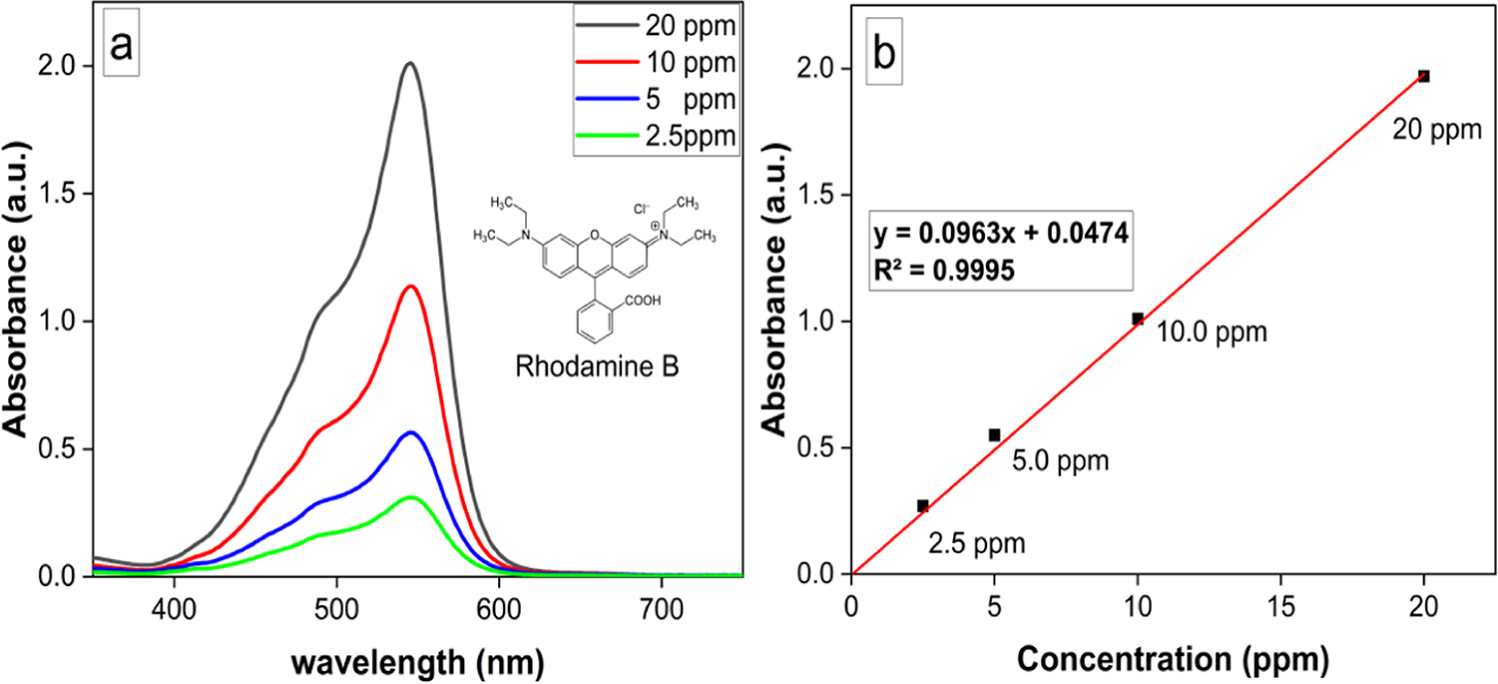
UV-Vis. spectrum of RB (**a**), Calibration curve at different concentrations of RB (**b**).

The RB removal was monitored spectrophotometrically at the absorbance maximum of RB dye viz. λmax = 546 nm which matched well with early-declared findings (Fig. 4a)^38^. The calibration curve of RB was performed using serial dilution (2.5, 5, 10, 15 and 20 ppm) of RB dye as illustrated in Fig. 4b.

**Fig. 5:**
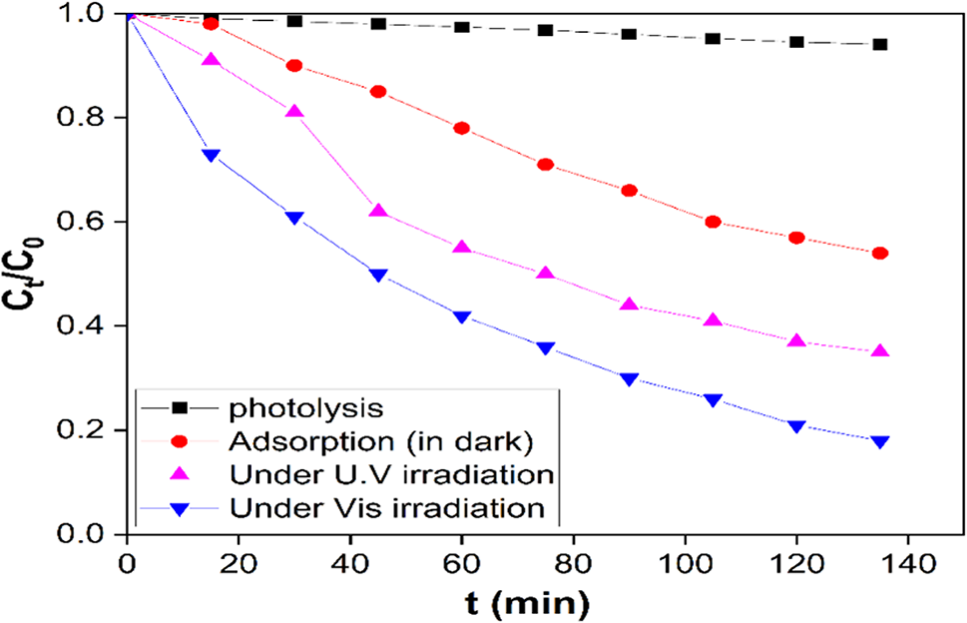
Removal of RB within 135 min due to photolysis (black line).

In this study, RB was selected as a model contaminant to evaluate the photocatalytic activity of the synthesized photocatalysts. The adsorption efficiency of RB by using AuNSTs under dark condition is represented in Fig. 5. The adsorption efficiency of RB without any light source was 40% after 135 min. After the adsorption, UV light was directed at the RB removal system containing the photocatalyst. The addition of AuNSTs into the RB solution under UV light irradiation demonstrated positive impact on RB removal efficiency after 135 min ( 62% removal). In this consideration, The comparison of the light absorption results between the dark and light irradiation conditions clearly demonstrated that most of the RB removal effects were due to photocatalytic degradation by the photocatalyst.

The AuNSTs demonstrated superior photocatalytic activity against RB, compared with RB photolysis without catalyst which chieved only 4.1% removal. On the other hand, the AuNSTs experienced RB removal by 82% in 135 min under Visible light irradiation^39^. The increase in photocatalytic activity by AuNSTs may be due to the increase in interfacial charge transfer efficiency for Au and due to enhancement in the charge pair separation and thereby inhibiting their recombination. AuNSTs demonstrated superior activity under visible spectrum with strong absorption at 685 nm.

#### Effect of pH on removal of RB

**Fig. 6:**
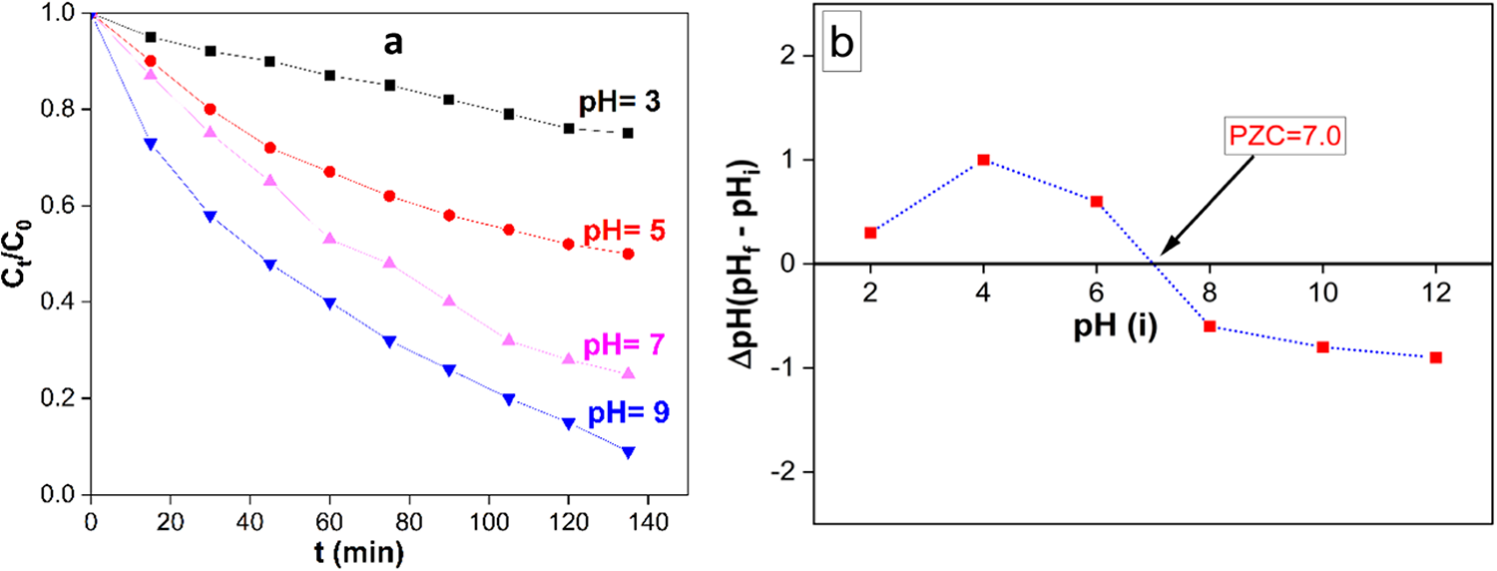
Removal (%) of RB with time at different solution pH (3.0, 5.0, 7.0 and 9.0) (10 mg of photocatalyst in 50 ml of 10 mg/l RB at 25 ^0^C) under visible light irradiation (**a**), Point of zero charges (PZC) of photocatalystat different pH values (**b**).

RB removal demonstrated high dependency on pH of the solution. The effect of initial pH values of RB solution was investigated for 90 min of specified experimental conditions (10 mg of the AuNSTs, 50 ml of 10 mg/L RB solution, Temp., = 25 ^0^C). The variation of RB removal (%) gradually with time at different solution pH (3.0–9.0) is represented in Fig. 6a. The maximum RB removal in equilibrium was observed at pH 9.0. Point of zero charges (PZC) of the AuNSTs was determined; 0.01 g AuNSTs was added to 50 mL (0.01 M NaCl solution). The pH values of the solutions were adjusted with HCl or NaOH to be as 2, 4, 6, 8, 10, and 12. The samples were continuously stirred at 200 rpm for 48 h. The pH values of the solutions were measured after the separation of AuNSTs. The pH of the PZC value was determined by using a plot that demonstrates the final pH versus the initial pH. The PZC was determined to be at pH = 7.0 (Fig. 6b).

It means that the surface charge of the AuNSTs are positive and negative when pH < PZC, and pH > PZC, respectively. Additionally, when the pH of the solution is equivalent to the pH of the PZC, the AuNSTs surface charge are neutral and the electrostatic force between the AuNSTs surface and ions (RB ions) is negligible^40^. The pH of the PZC of AuNSTs was 7.0, and this result could withstand the superior photocatalytic degradation of RB at pH 9.0 (Fig. 6a).

At thid pH value the net surface charge of the AuNSTs is Negative; consequently catalyst particles could attract the positive charge of RB and improves the photocatalytic degradation of RB. The photocatalytic degradation of RB began to decrease at pH = 5.0; this could be ascribed to the repulsive forces between the positive charge of RB and net surface charge of the AuNSTs which is positive at pH < 7.0.

#### Effect of initial RB concentration of RB

**Fig. 7:**
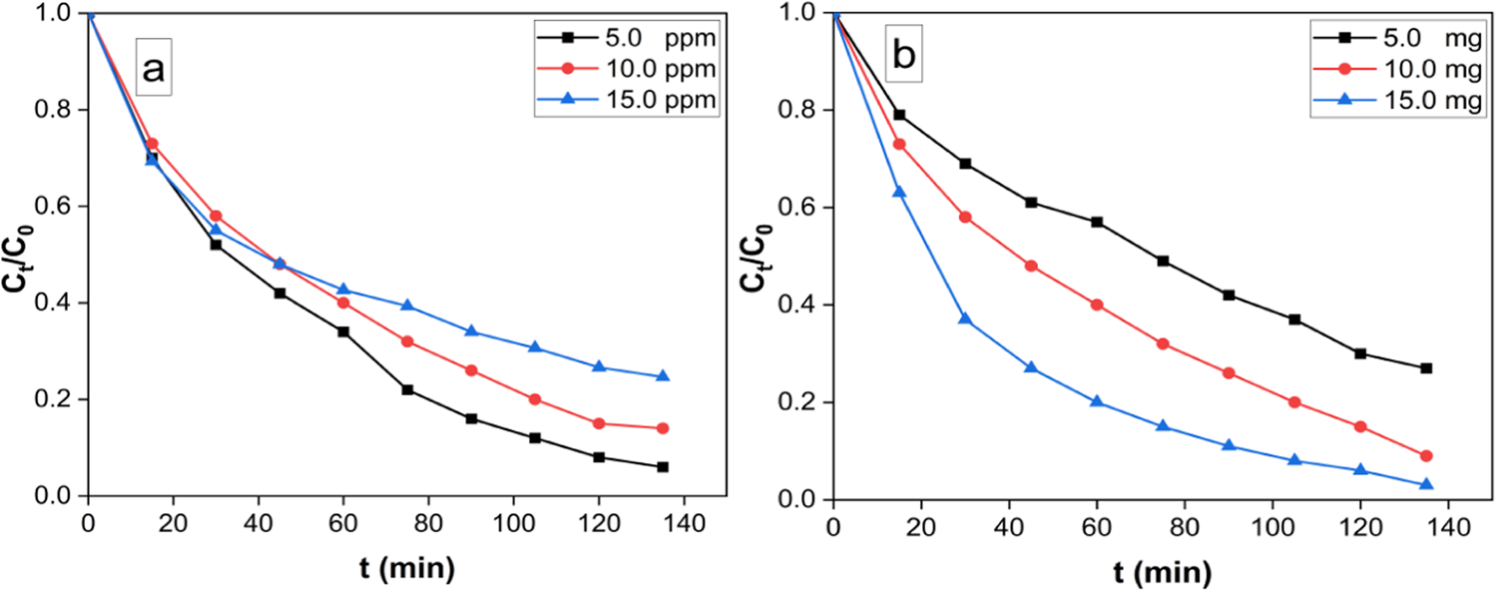
Removal % at different initial RB concentrations (5, 10, and 15 ppm), pH 9 and 10.0 mg nanocatalyst (**a**), Effect of the photocatalyst dose on the removal efficiency of RB (50 ml RB solution (10 ppm), T = 25 ^o^C and pH 9) under visible light irradiation (**b**).

As the initial RB concentration plays a vital role in the removal process, the effect of ionic strength of RB was investigated by varying the initial concentration of RB and keeping other reaction conditions unaltered. (Fig. 7a). The variation of removal % as a function of contact time at different initial RB concentration (5.0, 10.0, and 15.0 ppm) was investigated under Visible radiation. The results revealed that the degradation efficiency is inversely proportional to the concentration of RB.

#### Effect of gold nanostars dose on degradation efficiency

The impact of AuNST dose on the removal effeciency of RB under visible-light was investigated by varying the amount of photocatalyst between 5, 10, and 15 mg against a fixed concentration of RB (10 ppm) (Fig. 7b). The results indicated an increase in the removal efficiency upon increasing the photocatalyst dose from (5 to 15 mg). The observed increase in removal efficiency with photocatalyst amount, could be attributed to the increase in the available active area or active sites of the photocatalyst to volume ratio of RB solution^41^.

#### Kinetic studies

The degradation rate of pyridine was calculated using the following equation:

2 $$- ln{\text{ }}{C_t}/{C_O}= - Kt$$

**Fig. 8:**
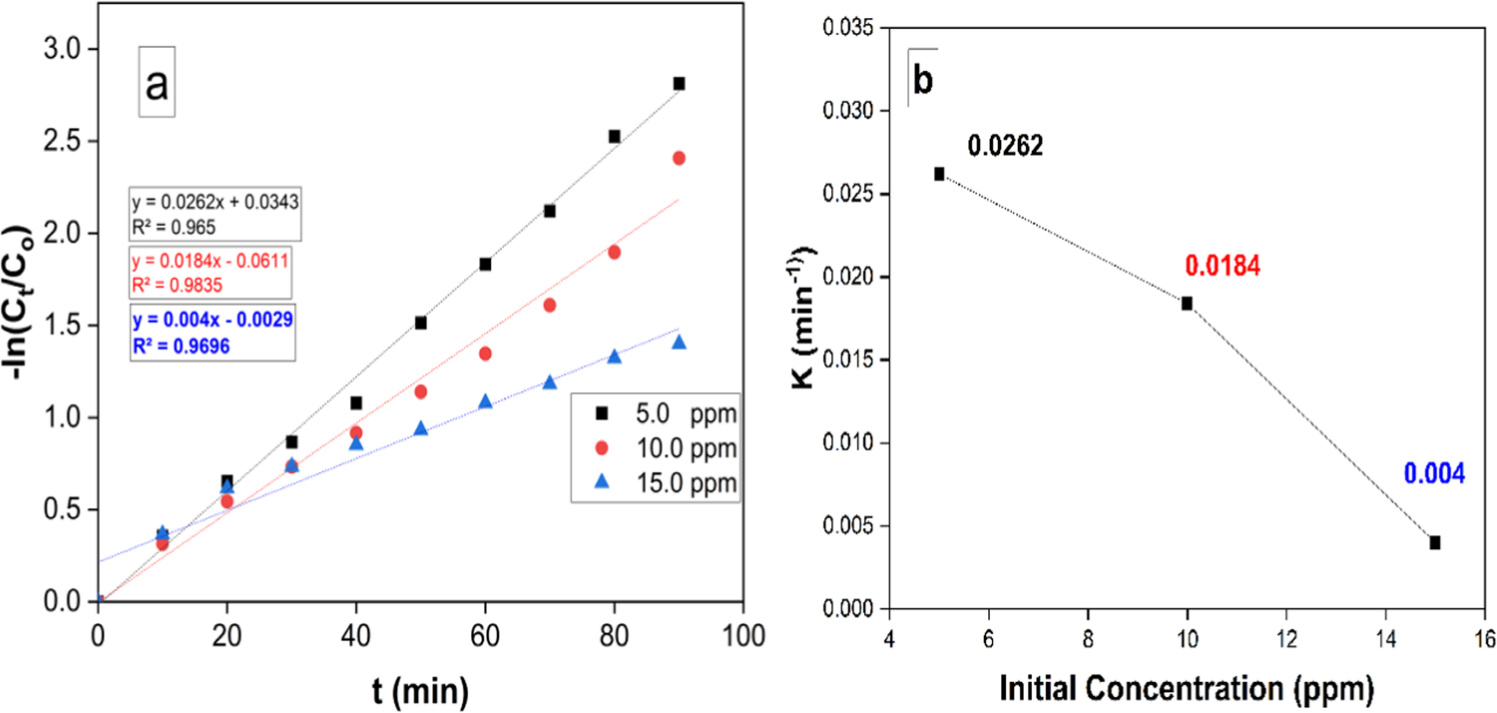
Pseudo-first-order reaction model for RB degradation under visible light irradiation and 10 mg catalyst, 50 mL of 5, 10, and 15 mg /L dye concentration (**a**), relation of apparent pseudo-first-order rate constants vs. initial RB concentration (**b**).

Where C_t_ and C_o_ are the remaining and the initial concentrations of RB respectively, while t is the removal time and k represents the removal rate constant. Figure 8a demonstrate the relation of - ln C_t_/C_o_ vs. t.

The results demonstrated that the removal reaction kinetics followed pseudo-first-order rate laws. Moreover, as revealed from (Fig. 8b), an increase of RB concentration could lead to the apparent pseudo-first-order rate constants to be decreased. This reliance on reaction rate constants on RB concentration is matched with presented literature^42–44^.

### Mechanism of photocatalytic activity

**Fig. 9:**
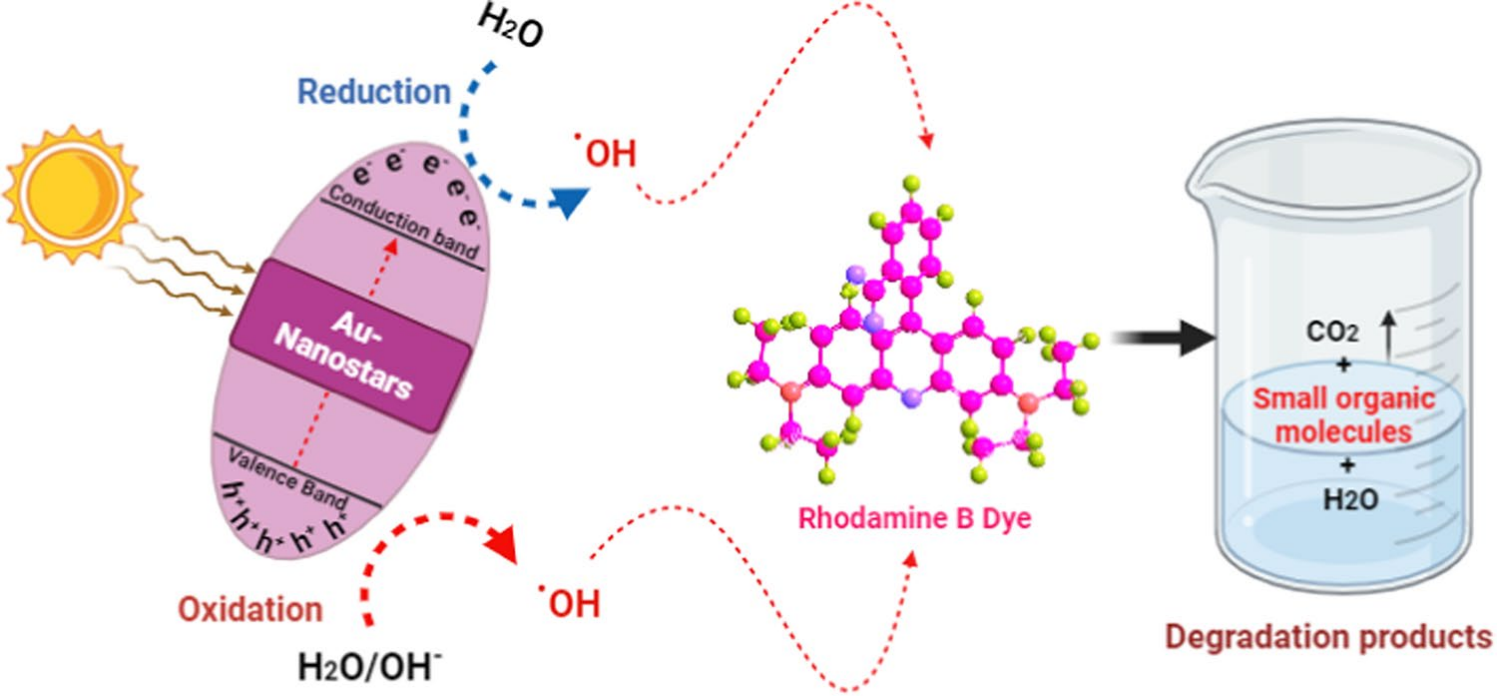
Proposed mechanism of photocatalytic degradation of RB by AuNSTs.

The potential mechanism can be elucidated as follows. Photodegradation mechanisms, which are influenced by variations in pH values, encompass the attack of hydroxyl radicals, explicit oxidation through the positive holes in the valence band, and explicit reduction by the electrons in the conduction band. In the presence of a photocatalyst, it is postulated that photocatalytic degradation is likely to occur due to the generation of electron-hole pairs on the surface of the employed photocatalyst induced by visible and UV irradiation^45,46^. The oxidative potential of these holes may either react with ^−^OH groups to form hydroxyl radicals or oxidize the reactive RB to generate a degradation product as represented in (Fig. 9).

The reactions involving RB and the employed photocatalyst can be summarized as shown in Eqs. (4–7).

4 $${\text{AuNSTs}}\,+\,{\text{hvAuNSts }}({{\text{e}}^{ - \,}}_{{{\text{CB}}}}+{{\text{h}}^{+\,}}_{{{\text{VB}}}})$$

5 $${{\text{h}}^{+\,}}_{{{\text{VB}}}}+{\text{ AuNSts AuNSt}}{{\text{s}}^+}\left( {{\text{Oxidation of the compound}}} \right)$$

Or

6 $${{\text{h}}^{+\,}}_{{{\text{VB}}}}+{\text{ O}}{{\text{H}}^ - }{\text{O}}{{\text{H}}^.}$$

7 $${\text{O}}{{\text{H}}^.}+{\text{ RB }}\left( {{\text{Degradation products}}} \right)$$

As AuNSts are subjected to visible irradiation, the excitation process could generate charge carriers; that could initiate redox reactions. Consequently, the resultant free radicals, including OH· and O_2_·^−^ could participate in the degradation of RB, with the formation of smaller organic compounds. Table 1 listed the photocatalytic activities of AuNPs and similar nanoparticles against different organic pollutants. The advanced photocatalytic performance of developed AuNSTs to different catalyst is tabulated in Table 1.

**Table 1:** Different nanomaterials with photocatalytic activities against different pollutants.

Photocatalyst	Targeted pollutant	Radiation/Light source	Degradation activity %	Time (min)	Refs.
TiO_2_ NPs	MO	UV	41.1	240	^47^
TiO2 NPs	RB	300 W xe arc lamp	34.1	150	^48^
AuNPs	MB	sunlight	87	20	^49^
AuNPs/porous GaN composite	MO	300 W Xenon lamp	67.5	300	^50^
AuNPs	MB	40 W Xenon, Philips	88	60	^51^
AuNPS	Eosin Y	UV	83.0	180	^52^
	Malachite green		65.0		
AgNPs	Eosin Y	UV	67.0	180	^52^
	Malachite green		64.0		
AuNSts	RB	Visible	94.0	135	This study

### Antimicrobial activity of synthesized AuNSTs Nanocatalyst

The in-vitro zone of inhibition (ZOI) test showed that AuNSts at a concentration of 20 µg/ml were more effective against *Staphylococcus aureus*, exhibiting a zone of inhibition measuring 23 mm and minimum inhibitory concentration (MIC) values of 0.625 µg/ml. Similarly, this formulation also displayed potent antimicrobial properties against *Escherichia coli*, yielding a ZOI of 20 mm and MIC values of 1.25 µg/ml, as presented in Table 2.

**Table 2:** Summarize In-vitro zone of inhibition ZOI (mm) and minimal inhibitory concentration MIC (µg/ml) of gold nanostars, against gram-positive and gram-negative bacteria.

Bacterial Strains	ZOI (mm) of AuNSts (20.0 µg/ml)	MIC (µg/ml) of AuNSts NPs	CN ZOI (mm)
*S. aureus*	23.0 ± 0.35	0.625	14.0 ± 0.40
*E. coli*	20.0 ± 0.54	1.25	19.0 ± 0.23

The experimental outcomes conclude that the fabricated AuNSts exhibited enhanced antimicrobial activity against Gram-positive bacteria relative to their effect on Gram-negative micro-organisms. This disparity in efficacy may arise from distinct structural and compositional characteristics inherent to the cell walls of Gram-negative versus Gram-positive bacteria^53,54^.

Gram-positive bacteria exhibit a thick layer of peptidoglycan, to which teichuronic and teichoic acids are chemically bonded. In contrast, Gram-negative bacteria possess a sparse covering of peptidoglycan, overlaid by an outermost layer composed of negatively charged lipopolysaccharides^53,55^. As aconsequence, the synthesized AuNSts exhibited a substantial inhibitory impact on Gram-positive bacterial species compared to Gram-negative bacterial species, as depicted in Table 2. The antibacterial activities of gold, silver and different similar nanoparticles were listed in Table 3.

**Table 3:** Antimicrobial efficacy of au, silver and similar nanoparticles against different pathogenic bacteria.

Nanocomposite	Method of synthesis	Particles size (nm)	Pathogen	ZOI (mm)	Ref.
Ag NPs	Chemical reduction method	35.50	*E.coli*	20 ± 1.5	^56^
ZnO NPs	precipitation method	24.84	*E.coli*	12.0	^57^
Ag-NPs	Green method	30.0	*K. pneumoniae*	25.5	^58^
			*B. cereus*	20.5	
Au NPs	Green method	20.0	*Klebsiella pneumonia*	10.33 ± 0.01	^59^
			*Streptococcus pneumonia*	15.33 ± 0.01	
Pt NPs	Laser ablation pulse	21.0	*S. mutans*	20.73 ± 0.10	^60^
ZnO @ TiO_2_	Laser ablation	34.0	*Bacillus cereus*	27.5	^61^
			*Proteus mirabilis*	24.0	
Pt-NPs	Two-step pulsed laser ablation in liquid (PLAL) technique	14.0	*E. faecium*	20.5 ± 0.30	^62^
			*K. pneumoniae*	16.5 ± 0.30	
AgNPs	Green method	100.8 ± 0.4	*S. aureus*	17 ± 1.00	^63^
			*P. aeruginosa*	24 ± 1.20	
Pd-NPs	Green method	24.20	*S. aureus*	18.3 ± 1.24	^64^
			*E.coli*	20.0 ± 0.81	
AuNSts	seed-mediated growth protocol	48.0	*E.coli*	20.0 ± 0.54	This study
			*S. aureus*	23.0 ± 0.34	

**Fig. 10:**
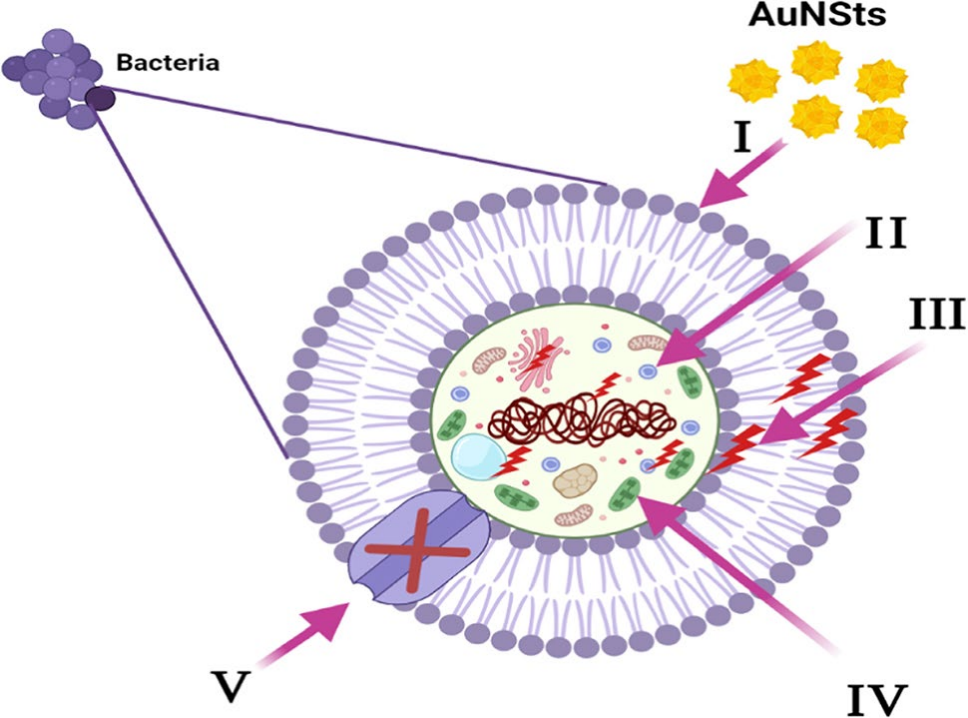
Schematic representation of the main pathways underlying the antibacterial potential of the AuNSts: (**I**) Adheres of AuNSts to and wrap the Bacterial cell surface, resulting in damage of bacterial cell. (**II**) AuNSts penetrate the microbial cells and affect the respective cellular machinery. (**III** AuNSts creates and increases ROS, leading to cell damage. (**IV**) AuNSts modulates the cellular signal system and causing cell death. (**V**) Finally, AuNSts block the ion transport from and to the microbial cells.

Figure 10 is a schematic of the possible antibacterial mechanism of AuNSTs^65–68^. AuNSts could initiate their process by attaching and wrapping themselves around the outside of the bacteria cells. This breaks down the membranes and changes the transport potential. AuNSts’s location inside the microbial cell separates all the structures inside, such as DNA, plasmids, and other important parts. After that, reactive stress caused by ROS production leads to cell death. Finally, AuNSts stop the movement of ions to and from microbe cells.

## Conclusion

Gold nanostars were successfully fabricated through a controlled seed–mediated growth (two stages protocol). The conducted nanostars were fully characterized and their impact on photocatalytic and antimicrobial applications was investigated. From the results, the particle size distribution analysis has been conduted for the obtained gold nano stars with average diameter of 48 ± 9 nm from tip to tip length. The AuNSts demonstrated notable photocatalytic efficiency in the removal of RB from aqueous solutions. AuNSTs demonstrated enhanced removal efficiency against rhodamine B dye (82.0 ± 0.35% in 135 min and pH 7) under visible irradiation. Remarkably, under conditions of pH 9 and utilizing 15 mg of AuNSts, approximately 94 ± 0.55% of a 10 ppm RB solution was effectively photodegraded after 135 min. In vitro assessments further corroborated the antimicrobial potential of AuNSts, as evidenced by zone of inhibition (ZOI) and minimum inhibitory concentration (MIC) results. Specifically, AuNSts exhibited significant antimicrobial activity against *E. coli* (ZOI: 20.0 ± 0.54 mm, MIC: 1.25 µg/ml) and *S. aureus* (ZOI: 23.0 ± 0.35 mm, MIC: 0.625 µg/ml). The synthesized AuNSts nanocatalyst holds promise for applications in antimicrobial treatments and wastewater purification processes. In the future work, we will determine the photocatalytic activity of the prepared composites in degradation of real wastewater samples containing mixed dyes and focused on the evaluation of the antibacterial activity of AuNSts against multidrug resistant bacteria and fungi for combating multi drug resistant crisis.

## Data Availability

Availability of data and material: The data presented in this study are available on request from the corresponding author.
